# Pharmacological and Predicted Activities of Natural Azo Compounds

**DOI:** 10.1007/s13659-016-0117-3

**Published:** 2017-01-04

**Authors:** Valery M. Dembitsky, Tatyana A. Gloriozova, Vladimir V. Poroikov

**Affiliations:** 1 National Scientific Center of Marine Biology, 17 Palchevsky Str., Vladivostok, Russia 690041; 2 Institute of Biomedical Chemistry, Moscow, Russia 119121

**Keywords:** Azo metabolites, Alkaloids, Fungi, Plant, Bacteria, Sponges, SAR

## Abstract

This paper describes research on natural azo compounds isolated from fungi, plant, bacteria, and invertebrates. More than 120 biologically active diazene containing alkaloids demonstrate confirmed pharmacological activity, including antitumor, antimicrobial, and antibacterial effects. The structures, origin, and biological activities of azo compounds are reviewed. Utilizing the computer program PASS, some structure–activity relationship new activities are also predicted, pointing toward possible new applications of these compounds. This article emphasizes the role of natural azo compounds as an important source of drug prototypes and leads for drug discovery.

## Introduction

Natural azo compounds are diazene containing compounds. Also called diimine or diimide, these metabolites have an azo moiety (–N=N–) [1–4]. The majority of natural diazene alkaloids have been isolated from microorganisms, plant parts (bark, berries, leaves, roots, and wood), fungi, fungal endophytes, lichenized ascomycetes and marine invertebrates [5–18].

Using the structure–activity relationships (SAR) approach realized in the computer program PASS, some additional activities were also predicted, indicating possible new applications for these compounds. Keeping in mind that presented below data on biological activity of azo metabolites characterize only a small part of possible biological potential in these molecules, we tried to estimate their biological activity spectra by computer prediction. For this purpose we used computer program PASS [19, 20], which predicts more than 7000 pharmacological activities, mechanisms of action, mutagenicity, carcinogenicity, teratogenicity and embryotoxicity on the basis of structural formulae of compounds. PASS predictions are based on SAR analysis of the training set consisting of more than 900000 of drugs, drug-candidates and lead compounds. Algorithm of PASS predictions is described in detail in several publications [21–24]. Using MOL or SD files as an input for PASS program, user may get a list of probable biological activities for any drug-like molecule as an output. For each activity Pa and Pi values are calculated, which can be interpreted either as the probabilities of a molecule belonging to the classes of active and inactive compounds respectively, or as the probabilities of the first and second kind of errors in prediction. Although the majority of the known biological activities for respective azo compounds are associated with antineoplastic action, their number is less than 60% among the predicted focal activities. A computer analysis of the predicted biological activity spectra showed that 58 types of biological activity are predicted with Pa > 70%, 199 with Pa > 50%, 463 with Pa > 30%, and 810 with Pa > Pi. This paper emphasizes the role of natural azo dyes as important sources for drug discovery.

## Azo Metabolites Derived from Actinomycetes and Fungal Species

**Fig. 1:**
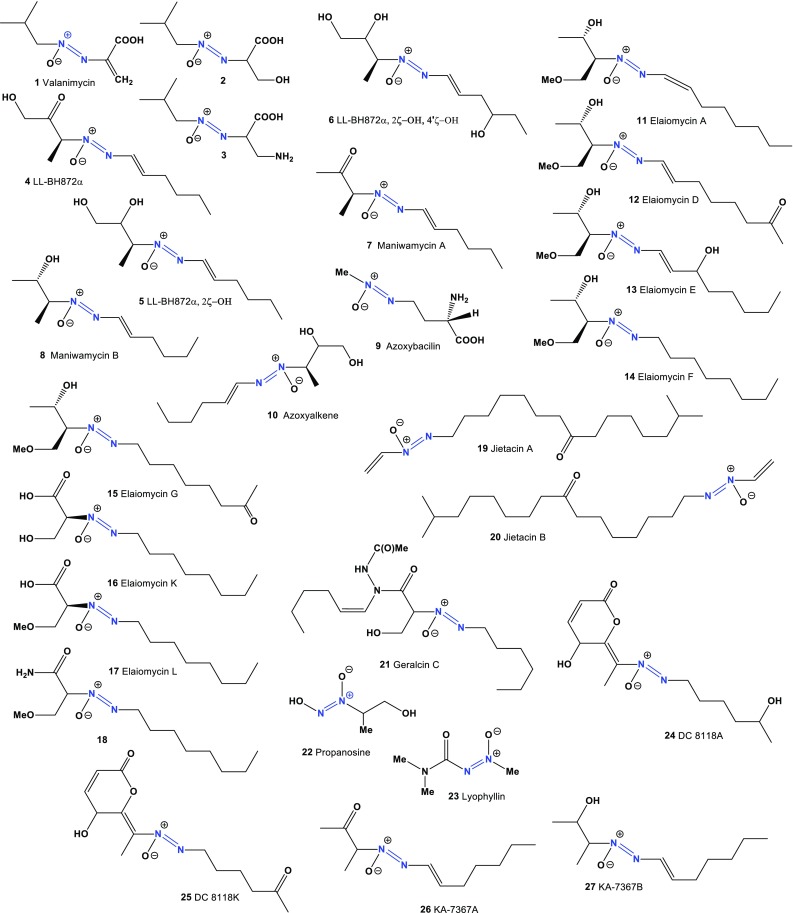
Biological active azo compounds derived from actinomycetes

Valanimycin (**1**), an azoxy antibiotic, was isolated from culture broths of *Streptomyces viridifaciens* MG456-hF10. It was active against both Gram-positive and Gram-negative bacteria, especially against *E. coli* BE1121, a DNA repair deficient mutant of *E. coli* K12. Valanimycin was toxic to in vitro cultures of cells of mouse leukemia L1210, P388/S, and P388/ADR, with IC_50_ values of 0.8, 2.7, and 1.4 pg/mL, respectively. It prolonged the life span of mice inoculated with Ehrlich carcinoma or L1210 [25]. Valanimycin derivative (**2**) was found in culture broth of a *S. viridifaciens* MG456-hF10 during biosynthesis of valanimycin (**1**) [26], and the elucidation of the structure was carried out on the more stable ammonia adduct (**3**) [27]. Predicted activities compounds (**1**–**10**) shown in Table 1 and structures shown in Fig. 1. α,β-Unsaturated azoxy-containing antibiotic LL-BH872α (**4**) was isolated from *Streptomyces hinnulinis* [28]. More recently, LL-BH872a, 2(*Z*)-OH (**5**) produced by *Actinomadura* sp., was isolated from the roots of *Prunus armeniaca* [27], and antibiotic LL-BH872a, 2(*Z*)-OH, 4′(*Z*)-OH (**6**), produced by *Streptomyces misionensis* [29].

**Table 1:** Confirmed and new biological activities of azo compounds (**1**–**10**) derived from actinomycetes

No.	Activity reviewed	Activities confirmed (Pa)	Additional predicted activities (Pa^a^)
**1**	Antibiotic antineoplastic	Antineoplastic (0.985) Antineoplastic antibiotic (0.848)	Phobic disorders treatment (0.819) Hepatic disorders treatment (0.662)
**2**	Not studied	–	Antineoplastic (0.880) Phobic disorders treatment (0.864) Hepatic disorders treatment (0.852)
**3**	Not studied	–	Phobic disorders treatment (0.907) Antiseborrheic (0.861) Antineoplastic (0.862)
**4**	Antibiotic	–	Hepatic disorders treatment (0.872) Antineoplastic (0.736) Antieczematic (0.733)
**5**	Antibiotic	Antibacterial (0.507)	Hepatic disorders treatment (0.819) Antiviral (arbovirus) (0.783) Antineoplastic (0.746)
**6**	Antibiotic	Antibacterial (0.527)	Hepatic disorders treatment (0.765) Antineoplastic (0.760) Antifungal (0.653)
**7**	Antibiotic antifungal	Antifungal (0.640)	Hepatic disorders treatment (0.778) Antineoplastic (0.763) Antieczematic (0.717)
**8**	Antibiotic antifungal	–	Phobic disorders treatment (0.860) Mucositis treatment (0.765) Antiviral (arbovirus) (0.747)
**9**	Antifungal	Antifungal (0.658)	Hepatic disorders treatment (0.793) Antiviral (arbovirus) (0.771) Antineoplastic (0.779)
**10**	Antifungal	Antifungal (0.632)	Hepatic disorders treatment (0.819) Antiviral (arbovirus) (0.783) Antineoplastic (0.746)

^a^Only activities with Pa > 0.5 are shown

Two antifungal antibiotics, maniwamycins A (**7**) and B (**8**), were isolated from the culture broth of *Streptomyces prasinopilosus*. Both antibiotics showed broad antifungal activities against *Candida albicans* IFM 40001, *C. albicans* N 508, *C. albicans* TIMM 0228, *C. albicans* TIMM 0237, *Cryptococcus neoformans* IFM 40038, *Nannizzia otae* JCM 1909, *Trichophyton mentagrophytes* IFM 40769, *T. mentagrophytes* IFM 40771, *T. rubrum* IFM 40768, and *Staphylococcus aureus* FDA 209P [30].

The microbial antifungal agent azoxybacilin (**9**) was isolated from the culture broth of *Bacillus cereus* NR2991. Azoxybacilin exhibits broad spectrum antifungal activity, especially against mycelial fungi, such as *Aspergillus fumigatus* and *Trichophyton mentagrophytes* [31, 32]. Azoxyalkene (**10**) is an unstable azoxy compound isolated from *Actinomadura* sp., an actinomycete growing in apricot roots. Preliminary biological assays revealed that exhibits weak antifungal activity against *Rhodotorula* sp. [27].

Elaiomycin (**11**) is an azoxy antibiotic that was first isolated from *Streptomyces hepaticus* and found to strongly inhibit the growth of *Mycobacterium tuberculosis* [33–36]. Elaiomycins D-G (**12**–**15**), antimicrobial and cytotoxic azoxides, were isolated from *Streptomyces* sp. HKI0708. Individual elaiomycins exhibit specific antimycobacterial, anti-*Aspergillus*, and cytotoxic activities, providing provisional data on SAR [37, 38]. Predicted activities compounds (**11**–**21**) shown in Table 2 and the structures shown in Fig. 1. Elaiomycins K (**16**), L (**17**) and amide elaiomycin K (**18**), azoxy-type antibiotics, were detected in the culture filtrate extract of *Streptomyces* sp. Tü 6399. Both metabolites show weak antibacterial activity against *Bacillus subtilis* and *Staphylococcus lentus* as well as against the phytophathogenic *Xanthomonas campestris* [39].

**Table 2:** Confirmed and new biological activities of azo compounds (**11**–**21**) derived from actinomycetes

No.	Activity reviewed	Activities confirmed (Pa)	Additional predicted activities (Pa^a^)
**11**	Antibiotic anti-mycobacterial	Antibacterial (0.489)	Hepatic disorders treatment (0.733) Antineoplastic (0.731) Antieczematic (0.697)
**12**	Antibiotic antifungal cytotoxic	Antifungal (0.646) Antineoplastic (0.738)	Hepatic disorders treatment (0.763) Vasodilator, peripheral (0.738)
**13**	Antibiotic antifungal cytotoxic	Antifungal (0.639)	Vasodilator (0.722) Antiinfective (0.684) Vasodilator (0.637)
**14**	Antibiotic cytotoxic	Antineoplastic (0.549)	Phobic disorders treatment (0.769) Antiviral (arbovirus) (0.688) Natural killer cell stimulant (0.637)
**15**	Antibiotic cytotoxic	Antineoplastic (0.599)	Hepatic disorders treatment (0.604) Vasodilator, peripheral (0.609)
**16**	Antibiotic antibacterial	–	Phobic disorders treatment (0.834) Antiviral (arbovirus) (0.805) Mucositis treatment (0.754)
**17**	Antibiotic antibacterial	–	Phobic disorders treatment (0.888) Preneoplastic (0.779) Mucositis treatment (0.756)
**18**	Antibiotic antibacterial	–	Phobic disorders treatment (0.837) Mucositis treatment (0.734) Natural killer cell stimulant (0.668)
**19**	Nematocide	–	Hepatic disorders treatment (0.849) Phobic disorders treatment (0.672) Antifungal (0.569)
**20**	Nematocide	–	Hepatic disorders treatment (0.849) Phobic disorders treatment (0.672) Antifungal (0.569)
**21**	Antibiotic antineoplastic	Antineoplastic (0.672)	Antiviral (arbovirus) (0.557)

^a^Only activities with Pa > 0.5 are shown

Nematocidal antibiotics, jietacins A (**19**) and B (**20**), isolated from the culture broth of a *Streptomyces* sp. [40, 41], exhibited 10 times higher activities against the pine wood nematode *Bursaphelenchus hgnicolus* in comparison to avermectin Bla, which is known to have a potent activity against various nematodes and which is used as a nematocidal agent in the veterinary field [42, 43].

**Fig. 2:**
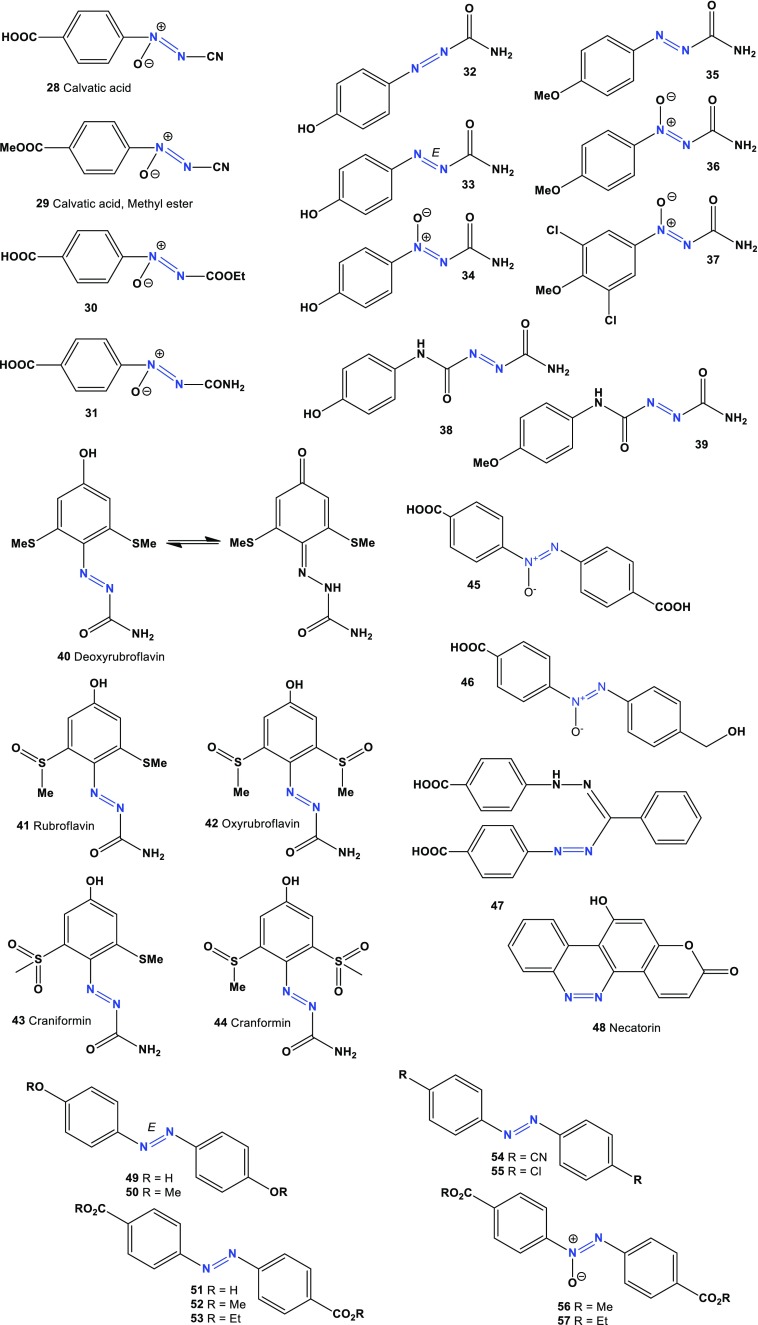
Aromatic azo compounds derived from actinomycetes and fungal species

Hydrazides, geralcin C (**21**) was isolated from *Streptomyces* sp. LMA-545 together with geralcins A, B, D and E. Geralcin C has exhibited an IC_50_ of 0.8 μM against KB and HCT116 cancer cell lines. Furthermore, geralcin C inhibited the *E. coli* DnaG primase, a Gram-negative antimicrobial target, with an IC_50_ of 0.7 μM [44]. The antibiotic propanosine (K-76, **22**), found in extracts of *Micromonospora chalcea* 671-AV2, has shown inhibitory activity against *Valsa ceratosperma* [45]. Predicted activities compounds (**22**–**39**) shown in Table 3 and the structures shown in Figs. 1 and 2.

**Table 3:** Confirmed and new biological activities of azo compounds (**22**–**39**) derived from actinomycetes

No.	Activity reviewed	Activities confirmed (Pa)	Additional predicted activities (Pa^a^)
**22**	Antibiotic antifungal	–	Antineoplastic (0.845) Phobic disorders treatment (0.775) Antiviral (picornavirus) (0.735)
**23**	Antibiotic	–	Antineoplastic (0.781) Phobic disorders treatment (0.750) Antiviral (arbovirus) (0.565)
**24**	Antibacterial antineoplastic	Antineoplastic (0.923) Antibacterial (0.613)	Antifungal (0.633) Genital warts treatment (0.648) Spasmolytic, urinary (0.605)
**25**	Antibacterial antineoplastic	Antineoplastic (0.927) Antibacterial (0.573)	Spasmolytic, urinary (0.687) Genital warts treatment (0.648) Immunosuppressant (0.596)
**26**	Antibiotic antifungal	Antifungal (0.640) Antibacterial (0.474)	Hepatic disorders treatment (0.778) Antineoplastic (0.763) Antieczematic (0.717)
**27**	Antibiotic antifungal	Antifungal (0.658) Antibacterial (0.514)	Hepatic disorders treatment (0.793) Antiviral (arbovirus) (0.771) Antineoplastic (0.779)
**28**	Antineoplastic antibiotic cytotoxic ornithine decarboxylase inhibitor	Anti-Helicobacter pylori (0.995)	Kidney function stimulant (0.636) Antieczematic (0.644) Preneoplastic conditions treatment (0.590)
**29**	Antineoplastic antibiotic	–	Anti-Helicobacter pylori (0.994) Preneoplastic conditions treatment (0.513)
**30**	Microtubule inhibitor	–	Anti-Helicobacter pylori (0.893) Antieczematic (0.751) Fibrinolytic (0.638)
**31**	Microtubule inhibitor	–	Anti-Helicobacter pylori (0.915) Preneoplastic conditions treatment (0.670) Kidney function stimulant (0.654)
**32**	Not studied	–	Antiseborrheic (0.793) Antiinflammatory (0.780) Hemostatic (0.668)
**33**	Nematocide	–	Antiseborrheic (0.793) Antiinflammatory (0.780) Hemostatic (0.668)
**34**	Nematocide	–	Anti-Helicobacter pylori (0.939) Antiseborrheic (0.717) Alopecia treatment (0.650)
**35**	Not studied	–	Antiinflammatory (0.714) Phobic disorders treatment (0.682) Preneoplastic conditions treatment (0.617)
**36**	Not studied	–	Anti-Helicobacter pylori (0.932) Preneoplastic conditions treatment (0.605)
**37**	Nematocide cytotoxic	–	Anti-Helicobacter pylori (0.932) Phobic disorders treatment (0.672) Preneoplastic conditions treatment (0.568)
**38**	Not studied	–	Apoptosis agonist (0.935) Antineoplastic (0.788) Alopecia treatment (0.653)
**39**	Not studied	–	Apoptosis agonist (0.920) Antineoplastic (0.724) Antiviral (arbovirus) (0.622)

^a^Only activities with Pa > 0.5 are shown

Another antibiotic, lyophyllin (**23**) was isolated from the mushroom *Lyophyllum shimeji* and showed inhibitory activity at a concentration of 50 μg/mL, inducing forebrain blisters within the cranial mesenchyme [46]. Two antibacterial and anti-tumoural agents, antibiotic DC1881A (**24**) and DC1881B (**25**) are produced by *Streptomyces* sp. DO-118 [47].

Two azoxy compounds, KA-7367A (**26**) and KA-7367B (**27**), which have antifungal activity, have been found in the culture broth of *Streptomyces* sp. (KC-7367, FERM BP-1277) [48]. Compound KA-7367A (**26**) showed antifungal activity against *Candida albicans*, *Aspergilus fumigatus*, *Cryptococcus neoformans*, *Trichophyton mentagraphytes*, and *T. rubrum*.

An antitumor antibiotic with a diazene N-oxide structure, calvatic acid (alvatic acid or calvatinic acid, **28**), and a methyl derivative (**29**), are produced by the fungi *Calvatia craniformis* [49] and *C. lilacina* [50] and from puffball mushrooms *Lycoperdon pyriforme* [51]. Calvatic acid inhibited the growth of Gram-positive and Gram-negative bacteria at a concentration of 3–6 μg/mL [50] and showed cytotoxic activity by inhibiting cultured Yoshida sarcoma cell growth [49], and it also displayed carcinostatic activity against hepatoma and K562 leukemia cells [52]. Calvatic acid also showed antibacterial activity against the Gram-negative, microaerophilic bacterium *Helicobacter pylori* [53]. Two calvatic acid analogues (**30** and **31**) have demonstrated anti-microtubular properties [54].

Azoformamide (**32**), its (*E*)-form (**33** and **35**), and its azoxy derivatives (**34** and **36**) were isolated from the puffball *Lycoperdon pyriforme* [55, 56]. Extracts of the basidiomycete *Lycoperdon pyriforme* yielded 4-methoxy-benzene-1-azoformamide (**33**) and 4-methoxy-benzene-1-ONN-azoxyformamide (**34**), which possess nematicidal activity against the parasitic nematode *Meloidogyne incognita*. The chlorinated derivative (**37**) is less active towards nematodes, but more cytotoxic compared to (**33** and **34**) [57]. Two azoxyformamides (**34** and **36**) and two azoformamide derivatives (**38** and **39**) were isolated from the fruiting bodies of *Calvatia craniiformis* and *Lycoperdon hiemale*, respectively. Compounds (**34**) and (**39**) showed radicle growth inhibitory activities against lettuce seedlings, suggesting that the azoxy moiety contributes to the inhibitory activity. The plant growth inhibitory activities of (**34**, **36**, and **39**) against barnyard millet seedlings were also reported [58]. The red minor pigment deoxyrubroflavin (**40**, activity see in Table 4) was isolated from the pufball mushroom *Calvatia rubro*-*flava* [59]. The orange pigment rubroflavin (**41**) was found in the dried fruit bodies of North American puffball *Calvatia rubro*-*flava* and in *C. craniformis* [59, 60]. Oxyrubroflavin (**42**), craniformin (**43**), and cranformin (**44**) were isolated from *C. rubro*-*flava* [59, 60].

**Table 4:** Confirmed and new biological activities of azo compounds (**40**–**58**) derived from actinomycetes and fungal species

No.	Activity reviewed	Activities confirmed (Pa)	Additional predicted activities (Pa^a^)
**40**	Not studied	–	Antiinflammatory (0.957) Antineoplastic (0.837) Hemostatic (0.822)
**41**	Not studied	–	Hemostatic (0.962) Antiinflammatory (0.956) Antineoplastic (0.808)
**42**	Not studied	–	Antiinflammatory (0.964) Hemostatic (0.952) Antineoplastic (0.833)
**43**	Not studied	–	Antiinflammatory (0.953) Antineoplastic (0.772) Hemostatic (0.577)
**44**	Not studied	–	Antiinflammatory (0.961) Antiarthritic (0.849) Antineoplastic (0.781)
**45**	Insecticide	–	Antieczematic (0.732) Kidney function stimulant (0.701) Preneoplastic conditions (0.677)
**46**	Insecticide	–	Immunosuppressant (0.647) Antieczematic (0.659) Fibrinolytic (0.606)
**47**	Not studied	–	Preneoplastic (0.692) Kidney function stimulant (0.631)
**48**	Not studied	–	Antimutagenic (0.758) Spasmolytic, urinary (0.699) Antineoplastic (0.660)
**49**	Not studied	–	Antiseborrheic (0.874) Phobic disorders treatment (0.784) Kidney function stimulant (0.752)
**50**	Not studied	–	Carminative (0.817) Phobic disorders treatment (0.786) Antiseborrheic (0.763)
**51**	Not studied	–	Antieczematic (0.843) Phobic disorders treatment (0.832) Kidney function stimulant (0.786)
**52**	Not studied	–	Phobic disorders treatment (0.840) Fibrinolytic (0.720) Preneoplastic conditions (0.703)
**53**	Not studied	–	Acaricide (0.821) Phobic disorders treatment (0.782) Antiseborrheic (0.773)
**54**	Not studied	–	Alopecia treatment (0.762) Phobic disorders treatment (0.777) Antiinflammatory, intestinal (0.699)
**55**	Not studied	–	Phobic disorders treatment (0.916) Antiseborrheic (0.802) Acaricide (0.726)
**56**	Not studied	–	Phobic disorders treatment (0.700) Preneoplastic conditions treatment (0.606) Immunosuppressant (0.612)
**57**	Not studied	–	Fibrinolytic (0.640) Acaricide (0.636)
**58**	Not studied	–	Mucositis treatment (0.831) Immunosuppressant (0.690)

^a^Only activities with Pa > 0.5 are shown

Two strains of the insect pathogenic fungus *Entomophthora virulenta* were found to produce a mixture of 4,4′-azoxybenzene dicarboxylic acid (**45**) and 4,4′-hydroxymethyl azoxybenzene carboxylic acid, which showed insecticidal activity (**46**) [61, 62]. A formazane derivative (**47**) has been isolated from *Agaricus silvicola* [63]. The mutagenic alkaloid necatorin (**48**) has been isolated from the mushroom *Lactarius necator* [64–66]. Two azo dyes, 4,4′-dihydroxyazobenzene (**49**) and its methyl derivative (**50**), have been identified in the fresh sporophores of the mushroom *Agaricus xanthodermus* [67]. Several azo dyes are fungal toxins (**51**–**57**) and are produced by entomogenous fungi, such as *Beauveria bassiana, Beauveria brongniartii, Metarhizium anisopliae,* and *Verticillium lecanii* [66, 68–72].

**Fig. 3:**
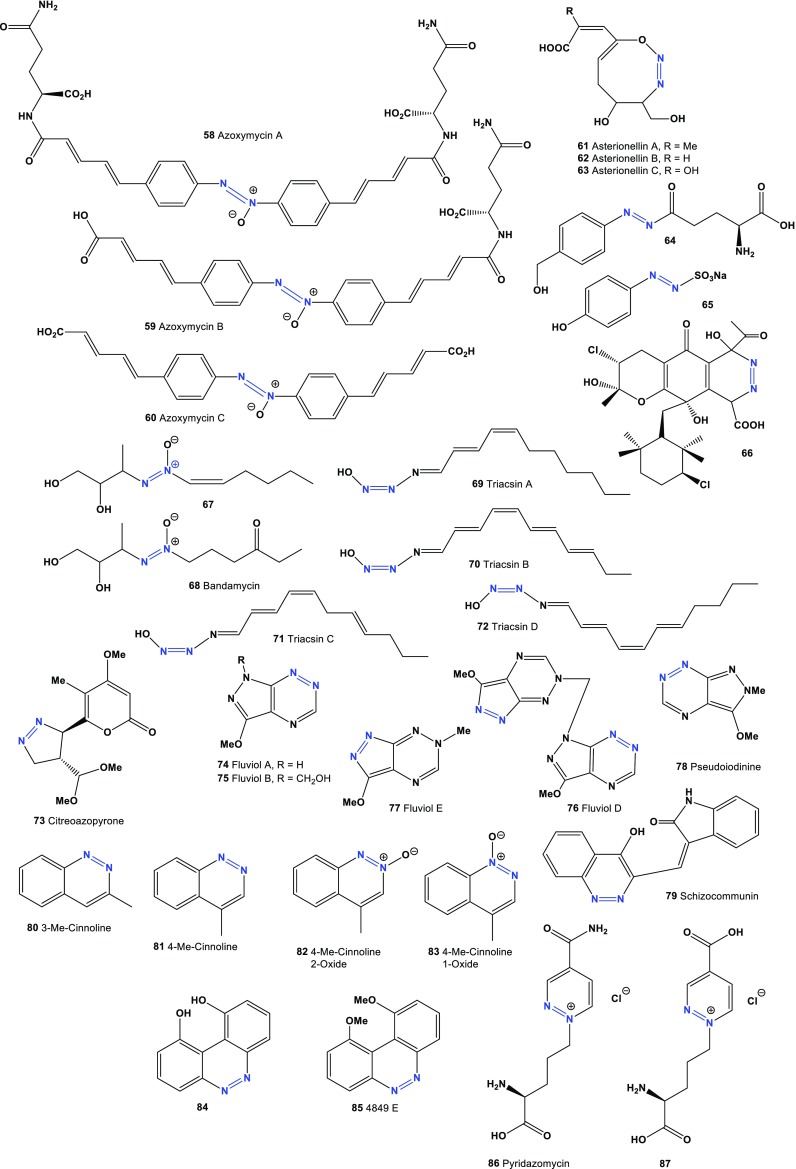
Miscellaneous azo compounds produced by actinomycetes and fungal species

Three novel aromatic azoxy compounds, azoxymycins A (**58**), B (**59**), and C (**60**), have been isolated and identified from *Streptomyces chattanoogensis* L10, and their biosynthetic pathways have been reported [73]. Predicted activities see in Table 5 and the structures shown in Figs. 2 and 3.

**Table 5:** Confirmed and new biological activities of azo compounds (**59**–**77**) derived from actinomycetes and fungal species

No.	Activity reviewed	Activities confirmed (Pa)	Additional predicted activities (Pa^a^)
**59**	Not studied	–	Mucositis treatment (0.776) Immunosuppressant (0.691)
**60**	Not studied	–	Antiviral (arbovirus) (0.694) Immunosuppressant (0.693) Antipsoriatic (0.625)
**61**	Not studied	–	Genital warts treatment (0.726) Antineoplastic (0.691)
**62**	Not studied	–	Genital warts treatment (0.726) Antineoplastic (0.704) Antiinflammatory (0.625)
**63**	Not studied	–	Genital warts treatment (0.726) Antileukemic (0.567)
**64**	Not studied	–	Mucositis treatment (0.761) Antiviral (arbovirus) (0.744)
**65**	Antibiotic antineoplastic	Antineoplastic (breast cancer) (0.552)	Alopecia treatment (0.641) Vascular (periferal) disease treatment (0.592)
**66**	Not studied	–	Antineoplastic (0.868) Antibacterial (0.678)
**67**	Antifungal	Antifungal (0.690)	Hepatic disorders treatment (0.994) Hepatoprotectant (0.786) Antiviral (arbovirus) (0.713)
**68**	Antifungal	Antifungal (0.662)	Hepatic disorders treatment (0.987) Antineoplastic (0.738)
**69**	Vasodilator Acyl CoA synthetase inhibitor	Vasodilator (0.881) Vasodilator, peripheral (0.599)	Antieczematic (0.830) Spasmolytic (0.678)
**70**	Acyl CoA synthetase inhibitor	Vasodilator (0.759)	Spasmolytic (0.649) Antineoplastic (0.668)
**71**	Acyl CoA synthetase inhibitor	–	Antieczematic (0.917) Vasodilator (0.901) Spasmolytic (0.706)
**72**	Acyl CoA synthetase inhibitor	–	Vasodilator (0.881) Antieczematic (0.830) Spasmolytic (0.678)
**73**	Antimicrobial antiviral antineoplastic	Antineoplastic (0.409)	Antiischemic, cerebral (0.752)
**74**	Antimicrobial antiviral antineoplastic	Antineoplastic (solid tumors) (0.618) Antineoplastic (renal cancer) (0.408)	Genital warts treatment (0.656) Cytostatic (0.562)
**75**	Antimicrobial antiviral antineoplastic	Antineoplastic (sarcoma) (0.482)	Gout treatment (0.865) Genital warts treatment (0.648)
**76**	Antimicrobial antiviral antineoplastic	–	Guanyl-specific ribonuclease T1 inhibitor (0.709) Genital warts treatment (0.531)
**77**	Antimicrobial antiviral antineoplastic	Antineoplastic (sarcoma) (0.469)	Anxiolytic (0.896) Psychotropic (0.745) Cognition disorders treatment (0.608)

^a^Only activities with Pa > 0.5 are shown

Asterionellins A (**61**), B (**62**), and C (**63**), eight membered compounds with an azoxy-like moiety, have been isolated from *Asterionella* sp. [74, 75]. The unstable agaritine derivative (**64**) and a metabolite (**65**) were extracted from the fruit-bodies of mushroom *Agaricus xanthoderma* [76]. Glutamylazophenol (**62**) was also found in *Agaricus* sp. [77]. Compound (**65**) has exhibited strong antibiotic and anticancer activities [77, 78].

It is known that the anti-infective agent azamerone, a meroterpenoid, isolated from the saline culture of marine-derived *Streptomyces* sp. Azamerone displays weak in vitro cytotoxicity against mouse splenocite populations of T cells and macrophages. A biosynthetic precursor azo compound (**66**) of azamerone has also been found in the same *Streptomyces* sp. [79]. The *Streptomyces* sp. Ank75 produced two azoxy antibiotics, **67** and **68**, and both compounds exhibited antifungal activity against *Candida albicans* and *Mucor miehei* [80].

Two vasodilators, designated WS-1228 A (triacsin C, **69**) and B (triacsin D, **70**), were discovered in the culture of *Streptomyces aureofaciens* [81, 82]. Four years later, Omura and co-authors [83] reported two triacsins A (**69**) and B (**70**), inhibitors of acyl-CoA synthetase, which were isolated from the cultured broth of *Streptomyces* sp. The structurally related compounds WS-1228 A and B, known to be hypotensive vasodilators, were also found to inhibit acyl-CoA synthetase. The four compounds have N-hydroxytriazene moiety in their structures in common. The IC_50_ values for triacsin A and WS-1228 A were 5.5 and 3.6 μg/mL, respectively. Triacsins A, B, C, and D, inhibitors of long chain acyl-CoA synthetase, possess different inhibitory potencies against the enzyme [84, 85]. Acyl-CoA synthetase activity in the membrane fraction of Raji cells was also inhibited by triacsins, which display the same hierarchy of inhibitory potency as that against the enzyme from other sources, that is, the inhibitory potency of triacsin C (**71**) is greater than that triacsin A, followed by that of triacsin D (**72**), and is greater than or equal to that of triacsin B [85].

A novel metabolite, citreoazopyrone (**73**), was isolated from the mycelium of *Penicillium citreo*-*viride*. It inhibited the growth of hypocotyls of lettuce seedlings [86]. A family of antibiotics named fluviols, which includes compounds (**74**, **75**), (**76**, **77**), and pseudoiodinine (**78**), are pyrazolo-[4,3-e]as-triazine derivatives, which are produced by *Pseudomonas fluorescens* var*. pseudoiodinum* and *Nostoc spongiaeforme*. All of these isolated compounds showed antimicrobial, antiviral, and antitumour activities [87–90]. Predicted activities compounds (**78**–**98**) shown in Table 6 and the structures shown in Fig. 3. Schizocommunin (**79**) was isolated from a culture of the fungus *Schizophyllum commune* and exhibited strong cytotoxicity against murine lymphoma cells [91].

**Table 6:** Confirmed and new biological activities of azo compounds (**78**–**97**) derived from actinomycetes and fungal species

No.	Activity reviewed	Activities confirmed (Pa)	Additional predicted activities (Pa^a^)
**78**	Antimicrobial antiviral antineoplastic	Antineoplastic (sarcoma) (0.413)	Atherosclerosis treatment (0.924) Genital warts treatment (0.600)
**79**	Cytotoxic antineoplastic	Antineoplastic (0.584) Antineoplastic (liver cancer) (0.797)	Endothelial growth factor antagonist (0.885) Angiogenesis inhibitor (0.632)
**80**	Not studied	–	Phobic disorders treatment (0.728) Antineurotic (0.685)
**81**	Not studied	–	Antineurotic (0.694) Phobic disorders treatment (0.648)
**82**	Not studied	–	Lysase stimulant (0.787) Kidney function stimulant (0.518)
**83**	Not studied	–	Lysase stimulant (0.787) Kidney function stimulant (0.518)
**84**	Interleukin 4 antagonist	–	Antiseborrheic (0.815) Kidney function stimulant (0.721) Phobic disorders treatment (0.749)
**85**	Antibacterial	–	Antineurotic (0.806) Phobic disorders treatment (0.752)
**86**	Not studied	–	Not predicted: MolCharge: 1
**87**	Not studied	–	Not predicted: MolCharge: 1
**88**	Cytotoxic	Antineoplastic (0.666) Antineoplastic (renal cancer) (0.614)	Pterin deaminase inhibitor (0.989) Natural killer cell stimulant (0.587)
**89**	Cytotoxic antifungal	–	Antiallergic (0.765) Cytostatic (0.712) Erythropoiesis stimulant (0.692)
**90**	Antineoplastic antileukemic	Antineoplastic (0.749) Antileukemic (0.622)	Genital warts treatment (0.936) DNA synthesis inhibitor (0.825) Cytostatic (0.701)
**91**	Antineoplastic antileukemic	Antineoplastic (0.752) Antileukemic (0.634)	Antimetabolite (0.938) DNA synthesis inhibitor (0.926) Neuroprotector (0.910)
**92**	Antibiotic	–	Antineoplastic (sarcoma) (0.730)
**93**	Not studied	–	Antineoplastic(0.768) Antibacterial (0.614) Antifungal (0.592)
**94**	Antimicrobial antiviral antineoplastic	Glycopeptide-like antibiotic (0.627) Antineoplastic(0.406)	Analgesic (0.637)
**95**	Antimicrobial antiviral antineoplastic	Glycopeptide-like Antibiotic (0.714) Antineoplastic (0.519) Antibacterial (0.409)	Analgesic (0.670)
**96**	Antimicrobial antiviral antineoplastic	Glycopeptide-like antibiotic (0.625) Antineoplastic(0.443)	Analgesic (0.601)
**97**	Antimicrobial antiviral antineoplastic	Glycopeptide-like antibiotic (0.713) Antineoplastic (0.547) Antibacterial (0.424)	Analgesic (0.641)

^a^Only activities with Pa > 0.5 are shown

**Fig. 4:**
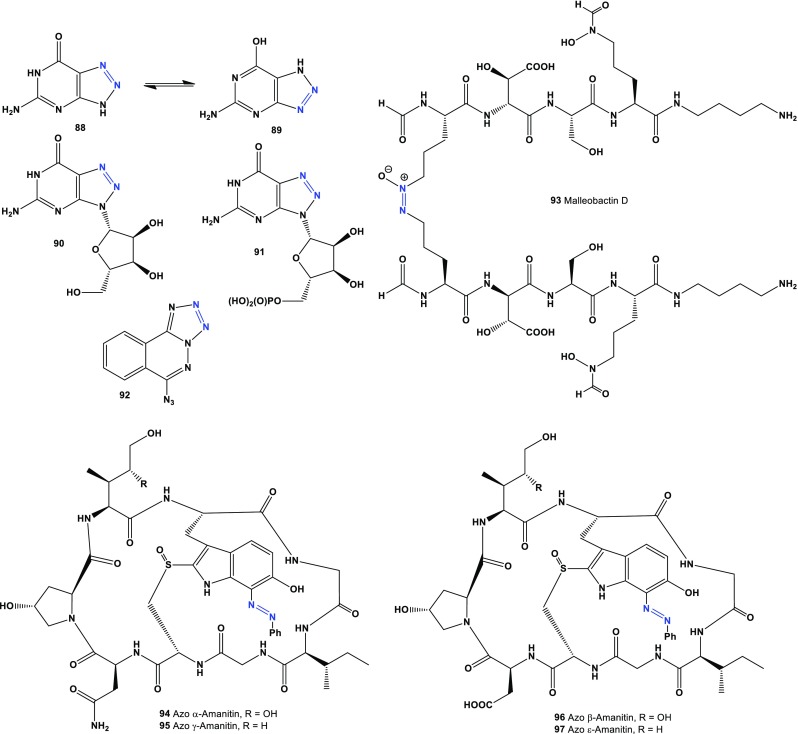
Biological active triazole derivatives, siderophores and octapeptides derived from actinomycetes and fungal species

3- and 4-methylcinnolines (**80** and **81**) were found in the volatile constituents of *Hibiscus esculentus* pods [92]. Azoxy compounds (**82** and **83**) were found in yeast extract [93]. The cinnoline derivatives (**84**) and 4849F (**85**) were isolated from a culture of *Streptomyces* sp. Compound (**84**) was shown to be an inhibitor of the IL-4 receptor, and alkaloid 4849F (**85**) has shown antibacterial activity [94]. Pyridazomycin (**86**), an antifungal antibiotic produced by *Streptomyces violaceoniger* sp. *griseofuscus*, inhibited the growth of *Mucor hiemalis* [95]. Pyridazomycin (**86**) and its analog (**87**), as chloride salts showed antimicrobial activity [96]. Compounds (**88** or **89**), also known as 8-azaguanine, is produced from guanine by *Spteptomyces albus* [97]. The cytotoxic effect of 8-azaguanine on the growth of carcinoma, sarcoma, osteogenic sarcoma, lymphosarcoma, and melanoma in animals was reported more than 65 years ago [98] (see Fig. 4).

Compound (**89**), also known as pathocidin, is an antifungal antibiotic that has been isolated from actinomycetes [99, 100] and inhibited the growth of many fungi, including *Penicillium chrysogenum*. 8-azaguanine-3*N*-β-d-ribofuranosyl (**90**) and 5′-phosphate-3*N*-β-d-ribofuranosyl (**91**) are known as natural metabolites and showed anticancer activity against L-1210 lymphoid leukemia and adenocarcinoma 755, among other activities [101]. A toxic red-tide dinoflagellate, *Gymnodinium breve*, produced the antibiotic 6-azidotetrazolo[5,1-a]phthalazine (**92**) [102].

The *Burkholderia* species secretes a variety of extracellular enzymes with proteolytic, lipolytic, and hemolytic activities. Several strains also secrete toxins, antibiotics, and siderophores [103]. The unusual dimeric siderophore, malleobactin D (**93**), was isolated from *Burkholderia pseudomallei* [104].

The amatoxins are a group of bicyclic octapeptides produced by some species of mushrooms belonging to the Agaricales: *Amanita phalloides, A. ocreata, A. verna, A. bisporigera, Conocybe filaris, Galerina marginata, G. venenata, Lepiotia castanea, L. helveola, L. subincarnata*, *L. brunneoincarnata, L. brunneolilacea*, and close relatives. Selected amatoxins showed toxicity to heat, the digestive tract, and strong inhibition of RNA polymerase II [105–108]. Azo-amanitins (**94**–**97**) are semi-natural compounds, and they showed antiviral, antimicrobial, and anticancer activities [109–112]. Predicted activities compounds (**88**–**97**) shown in Table 6 and the structures shown in Fig. 3.

## Azo Metabolites Derived from Terrestrial and Marine Sources

**Fig. 5:**
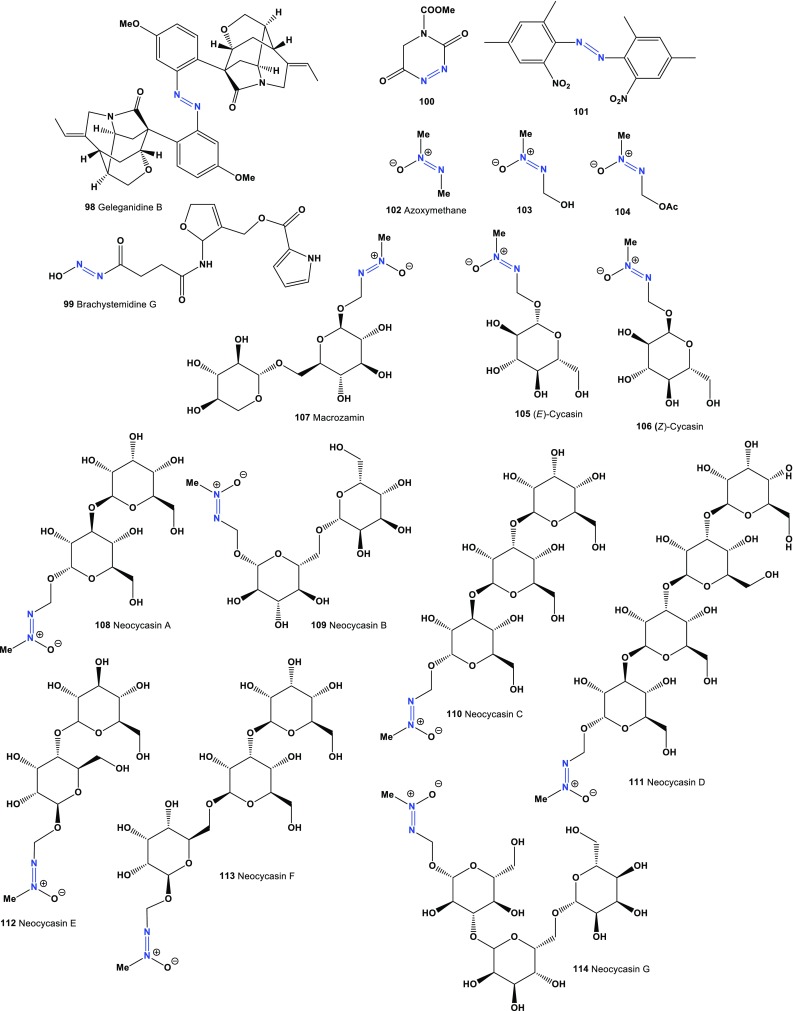
Novel biological active azo compounds derived from plants

**Fig. 6:**
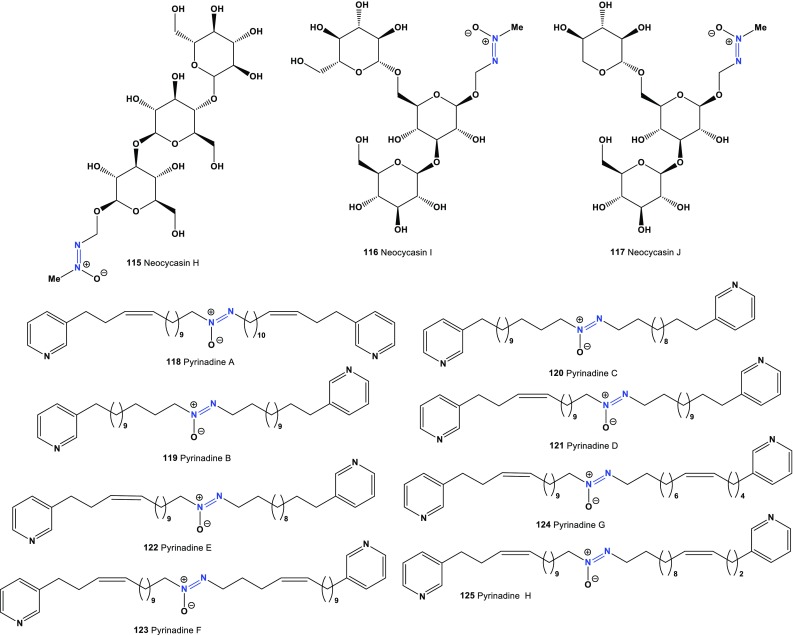
Bioactive azoxy-glycosides derived from Cycadaceae plants and pyridine derivatives produced by marine sponge

The novel dimeric monoterpenoid indole alkaloid, geleganidine D (**98**), was isolated from the roots of flowering plant *Gelsemium elegans*. It showed moderate cytotoxic activity against MCF-7 and PC-12 cells [113]. Predicted activities compounds (**98**–**117**) shown in Table 7 and the structures shown in Figs. 5 and 6.

**Table 7:** Confirmed and new biological activities of azo compounds (**98**–**117**) derived from plants

No.	Activity reviewed	Activities confirmed (Pa)	Additional predicted activities (Pa^a^)
**98**	Cytotoxic	Antineoplastic (0.730)	Antiprotozoal (plasmodium) (0.641) Antiprotozoal (0.579)
**99**	Immunosuppressant	–	β-1,3-galactosyl-O-glycosyl-glycoprotein β-1,6-N-acetylglucosaminyl transferase inhibitor (0.954) Antineoplastic (0.609)
**100**	Not studied	–	Neurodegenerative diseases treatment (0.920) Antiparkinsonian (0.900) Anxiolytic (0.796)
**101**	Not studied	–	Acaricide (0.721) Antiviral (arbovirus) (0.681)
**102**	Not studied	–	Not predicted: MolCharge: 1
**103**	Not studied	–	Not predicted: MolCharge: 1
**104**	Not studied	–	Antiinfertility, female (0.940) Antineoplastic(0.835) Phobic disorders treatment (0.726)
**105**	Toxic carcinogenic mutagenic neurotoxic	Carcinogenic (0.975) Toxic (0.932) Neurotoxic (0.746)	Embryotoxic (0.957) Teratogen (0.952) Hepatotoxic (0.716)
**106**	Toxic carcinogenic mutagenic neurotoxic	Carcinogenic (0.975) Toxic (0.932) Eurotoxic (0.746)	Embryotoxic (0.957) Teratogen (0.952) Hepatotoxic (0.716)
**107**	Not studied	–	Antineoplastic (0.892) Genital warts treatment (0.870) Antiinfective (0.837)
**108**	Toxic carcinogenic mutagenic neurotoxic	Carcinogenic (0.964) Toxic (0.943) Neurotoxic (0.822)	Embryotoxic (0.960) Teratogen (0.950) Hematotoxic (0.695)
**109**	Not studied	–	Genital warts treatment (0.876) Antineoplastic (0.866) Vasoprotector (0.851)
**110**	Not studied	–	Antineoplastic (0.892) Genital warts treatment (0.870) Antiinfective (0.837)
**111**	Not studied	–	Antineoplastic (0.892) Genital warts treatment (0.870) Antiinfective (0.837)
**112**	Not studied	–	Antineoplastic (0.897) Genital warts treatment (0.870) Antiinfective (0.837)
**113**	Not studied	–	Antineoplastic (0.889) Genital warts treatment (0.857) Vasoprotector (0.828)
**114**	Not studied	–	Antineoplastic (0.889) Genital warts treatment (0.857) Vasoprotector (0.828)
**115**	Not studied	–	Antineoplastic (0.900) Genital warts treatment (0.864) Hepatic disorders treatment (0.791)
**116**	Not studied	–	Antineoplastic (0.889) Genital warts treatment (0.857) Vasoprotector (0.828)
**117**	Not studied	–	Antineoplastic (0.889) Genital warts treatment (0.842) Hepatic disorders treatment (0.810)

^a^Only activities with Pa > 0.5 are shown

Alkaloid brachystemidine G (**99**) was isolated from the roots of *Brachystemma calycinum*. This compound is a potent immunosuppressive agent, as demonstrated by its inhibition of mouse T and B-lymphocyte proliferation, with IC_50_ value of 5.6 μg/mL [114]. The 1,2,4-triazine derivative (**100**) was extracted from the seeds of the tropical flowering plant *Butea monosperma* [115]. It is known that the odor of this plant kills mosquitoes, the flowers are used as a dyeing color, and the gum, called kamarkas (Hindi), is used in food dishes [116]. Alkaloid (**101**) was isolated from the leaf extract of the flowering plant *Aconnitum sungpanense* [117].

Azoxy-glycosides have a common aglycone, methylazoxymethanol (MAM) and are found in Cycadaceae plants. To date, all of these glycosides that have been isolated have β-glycosidic linkages [118]. Methyl-azoxymethane (**102**), methylazoxy-methanol (MAM, **103**), methylazoxymethanol acetate (**104**), and cycasin (**105** and **106**) metabolites were extracted from the seeds and roots of cycad plants Cycadaceae, Stangeriaceae, and Zamiaceae [16, 118–123], which are conifers common to the tropics and subtropics. MAM (**103**) was shown to induce a variety of tumors, primarily liver and renal cell carcinomas [124]. Cycasins (**105** and **106**) and macrozamin (**107**) are very toxic azoxyglycosides of Cycadales.

Azoxy-glycosides may have played an important ecological role as antiherbivore defenses. Cycasin, which together with macrozamin represent the major azoxy-glycosides occurring in cycads, has been reported to elicit responses similar to those that have been observed during carcinogenicity, mutagenicity, and neurotoxicity assays. The first isolation of a glycoside, neocycasin A (**108**), was reported [125]. More recently, a range of neocycasin compounds, including neocycasin B, C, D, E, F, G, H, I, and J (**109**–**117**), were isolated from different plants [126–133].

The first identified cytotoxic *bis*-3-alkylpyridine alkaloid containing an azoxy moiety, pyrinadine A (**118**), was isolated from an Okinawan marine sponge *Cribrochalina* sp. [134]. Additional cytotoxic *bis*-3-alkylpyridine alkaloids, pyrinadines B, C, D, E, F, G, and H (**119**–**125**) were isolated from the same Okinawan marine sponge. Pyrinodemins showed cytotoxicity against P388 murine leukemia cells [135, 136]. Predicted activities compounds (**118**–**125**) shown in Table 8 and the structures shown in Fig. 6.

**Table 8:** Confirmed and new biological activities of azo compounds (**118**–**125**) derived from marine sponge

No.	Activity reviewed	Activities confirmed (Pa)	Additional predicted activities (Pa^a^)
**118**	Cytotoxic	Antineoplastic (0.776)	Antieczematic (0.693) Antiinflammatory (0.646)
**119**	Cytotoxic	Antineoplastic (0.747)	Cardiovascular analeptic (0.567) Fibrinolytic (0.538)
**120**	Cytotoxic	Antineoplastic (0.747)	Cardiovascular analeptic (0.567) Fibrinolytic (0.538)
**121**	Cytotoxic	Antineoplastic (0.771)	Antieczematic (0.671) Antiinflammatory (0.645)
**122**	Cytotoxic	Antineoplastic (0.771)	Antieczematic (0.671) Antiinflammatory (0.645)
**123**	Cytotoxic	Antineoplastic (0.771)	Antieczematic (0.671) Antiinflammatory (0.645)
**124**	Cytotoxic	Antineoplastic (0.771)	Antieczematic (0.671) Antiinflammatory (0.645)
**125**	Cytotoxic	Antineoplastic (0.776)	Antieczematic (0.693) Antiinflammatory (0.646)

^a^Only activities with Pa > 0.5 are shown

## Concluding Remarks

Natural azo metabolites comprise a rare group of natural products. They are primarily present in fungi, plant, and microorganisms have also been detected in some invertebrates. Little information is known about the biological activities of these metabolites. Nevertheless, reported activities for these isolated compounds have shown strong anticancer, antibacterial, antiviral, and other activities. The widest spectra of biological activities are exhibited by isolated azo metabolites. Natural azo compounds have been shown to be promising candidates for the development of new drugs used for the treatment of several diseases.
